# Practical First Principles

**Published:** 1902-07

**Authors:** 


PRACTICAL FIRST PRINCIPLES. Simplifying the study of normal and abnormal structure and function, and aiding diagnosis. Designed for the use of students and practitioners of medicine. By A. H. P. LEUF, M. D., associate editor of The Medical Council, Philadelphia. Small 8vo, 105 pages, nearly 50 illustrations. 1901. [Price, $1.00 net.]

That is the full title page of the weirdest, wooziest work which has wobbled forth into a chilly and unfeeling world in many a moon. There is a preface—of course—and one paragraph will be sufficient: "The illustrations are almost all original and diagramatic, the former with a view to avoiding marked connection with any given text-book, and the latter so as to impress a principle upon the reader's mind rather than a set picture which he never sees for the reason that it does not exist."

That alone is enough to Peleeize the book—it is a book because it has a number of pages and a front and a back cover. There has been a growing suspicion that in these days of color work, photo-engraving, we were outgrowing the diagramatic idea, but Dr. Leuf doesn't believe so along with the rest of us. This is shown most strikingly in his illustrations, one of which, the "schematic representation of a liver lobule," looks as much like an old-fashioned poor house pine coffin as it does like a pair of papa's pants cut down for Willie; and you can take your choice according to whether you are cold-hearted or merely sympathetic.

In the effort to hew close to the line of originality, letting the chips fall where they may, and "avoid marked connection with any text-book," great and complete success has been achieved. The illustrations are not like those in any other text-book in the world. Montgomery, of Texas, is the original offender, for according to the author's opening words in Chapter I., Montgomery, of Texas, in 1881, asked: "Are we cell aggregates?" That plaintive cry from the place where they raise cattle and hell, was the beginning of all this trouble. The second sentence of the first chapter is: "There can be no doubt of it!" That should have been enough. It answered Montgomery's question, and it should have been the end of the book. The publishers have paid Dr. Leuf a pretty compliment in sending forth his book, but they have done him a grievous wrong and incidentally wasted 106 pages of fair book paper and a couple of deep blue covers. It isn't big enough to be in the way and it's too little to stand alone. So, what's the use?

